# ﻿A new species of *Aulacaspis* and a revived combination of Diaspididae (Hemiptera, Coccomorpha) from China

**DOI:** 10.3897/zookeys.1174.105851

**Published:** 2023-08-15

**Authors:** Minmin Niu, Bo Cai, Jiufeng Wei

**Affiliations:** 1 College of Plant Protection, Shanxi Agricultural University, Taigu, 030801, China Shanxi Agricultural University Taigu China; 2 Post-Entry Quarantine Station for Tropical Plant, Haikou Customs District, No. 9 West Haixiu Road, Haikou, 570311, China Post-Entry Quarantine Station for Tropical Plant Haikou China

**Keywords:** Armored scale insect, molecular phylogenetic analysis, new record, pest, taxonomy

## Abstract

A new species of armored scale insect, *Aulacaspisfanjingshanensis***sp. nov.** is described and illustrated based on adult female specimens collected on Rosaceae plants in China. A key to the *Aulacaspis* species known from Guizhou Province of China is provided. Our molecular study suggests that *Aulacaspisschizosoma* (Takagi, 1970) is not a true member of the genus *Aulacaspis*; the genus *Superturmaspis* Chen, 1983 is revived and *A.schizosoma* is transferred to it as *Superturmaspisschizosoma* (Takagi, 1970), **revived combination**, based on a molecular phylogeny.

## ﻿Introduction

Diaspididae, the largest family of Coccomorpha with more than 2700 species (García Morales et al. 2016), includes important economic pests and is one of the most invasive insect groups in the world.

The genus *Aulacaspis* Cockerell, 1893 belongs to the Chionaspidina, within the tribe Diaspidini, in the subfamily Diaspidinae (Normark et al. 2019). They are generally found living in groups on the host plants and suck the host plants’ sap through their piercing mouthparts. At high infestation levels of the pests, the leaves and branches of the host plants often wither, or even die, seriously affecting the yield and quality of the host plant, causing greater economic losses.

One hundred and fifty-one species of *Aulacaspis* have been recorded in the world and distributed worldwide (García Morales et al. 2016). The genus was established by Cockerell in 1893 based on *Aspidiotusrosae* Bouché, 1833, as the type species. This genus has been accepted by other scholars for more than 20 years (Kuwana 1926; Ferris 1936). Currently, the body shape of *Aulacaspis* mainly includes two types: the ‘*rosae*-type’ and the ‘*vitis*-type’ (Takagi 2012). And arrangement and number of dorsal and marginal ducts and median lobes are considered important morphological taxonomic features of *Aulacaspis*.

Guizhou Province in China has a subtropical monsoon climate, with rich species resources (http://invest.guizhou.gov.cn/tzgz/tzgk/). Hitherto, seven *Aulacaspis* species are currently recorded in Guizhou Province, namely *A.crawii* (Cockerell), *A.guiyangensis* Tian & Xing, *A.longanae* Chen, Wu & Su, *A.paralonganae* Tian & Xing, *A.saigusai* Takagi, *A.yabunikkei* Kuwana and *A.zunyiensis* Wei (Easton and Pun 1999; Hua 2000; Wei et al. 2016; García Morales et al. 2016; Tian and Xing 2022).

*Aulacaspis* was placed in Chionaspidina by Takagi (1999), within the tribe Diaspidini, in the subfamily Diaspidinae. Normark et al. (2019) found the non-monophyly of a genus-level phylogeny and taxonomy of Chionaspidina, including the *Aulacaspis* and *Chionaspis* Signoret, 1868.

Recently, a new species of *Aulacaspis* was discovered in Guizhou Province of China, and it is described and illustrated herein. And molecular study revealed the need for the resurrection of the genus *Superturmaspis* Chen, which was synonymized with *Aulacaspis* by Takagi (1985, 1999).

## ﻿Materials and methods

### ﻿Taxonomy

Samples of plants infested by the new species described in this study were collected in Fanjing Mountain (Guizhou Province, China). Permanent slide mounts of adult females from the samples were made according to Henderson (2011). Illustrations of adult females of the new species were drawn based on the type-series of slide-mounted specimens, each showing an overview of the dorsum on the left side and the venter on the right. Enlarged details of the significant features are illustrated around the body, not in direct proportion to each other. The morphological terminology and measurements in the descriptions follow those of Miller and Davidson (2005). All measurements are given in micrometres (µm). Measurements were made using the measurement tools NIS-Elements D. The type-series of the new species is deposited in the Insect Collection of Shanxi Agricultural University, Taigu, Shanxi Province, China (SXAU) and the Entomological Museum, Northwest A&F University, Yangling, Shaanxi, China (NWAFU).

### ﻿Molecular phylogenetic analysis

#### ﻿DNA extraction and amplification

Each specimen was subjected to a joint molecular/morphological preparation protocol that resulted in genomic DNA from a single specimen and a permanent slide-mount of its cuticle (Normark et al. 2019). We used the single adult whole bodies for DNA extraction from each collection site. The total DNA was extracted using the Qiagen DNeasy Blood & Tissue kit (Qiagen, Valencia, California, USA), stored at 4 °C in the refrigerator. Other parts of the extraction follow the manufacturer’s protocols of the Qiagen kit. Polymerase chain reaction (PCR) was performed to amplify regions of the nuclear protein-coding gene elongation factor-1 alpha (EF-1α) the D2 and D3 expansion segments of the large subunit ribosomal RNA (28S). The PCR reactions contained a total of 25 μl, which included 12.5 μl 2×Taq MasterMix (with dye) (Coolaber, Beijing, China), 8.5 μl distilled water, 1 μl of each primer and 2 μl DNA template, and a negative control was included for all reactions. To amplify the fragment, we used the T100TM Thermal Cycler (BIO-RAD Laboratories, Inc., Hercules, California) to execute the cycling profiles given in Table 1.

**Table 1.** PCR primers and annealing temperatures, from Gruwell et al. (2007), Andersen et al. (2010) and Normark et al. (2019). Before thermal cycling begins, all PCR reactions start with a single 2-minute denaturation at 95 °C. Each subsequent cycle consists of a 30 s denaturation at 95 °C, a 1-minute annealing step with temperature given below, and a 2-minute extension at 72 °C. After thermal cycling is completed, all PCR reactions end with a single 10-minute extension at 72 °C. Primer sequences are given from 5' to 3'.

Gene Region	Forward Primer	Reverse Primer	Annealing temperature profile
28S	28s_s3660	28s_a335	51 °C, 35 cycles at 48 °C
	GAG AGT TMA ASA GTA CGT GAA AC	TCG GAR GGA ACC AGC TAC TA	
EF-1α	EF-1α(a) (amplification/sequencing) GAT GCT CCG GGA CAY AGA G	EF2rod (amplification/sequencing) ATG TGA GCG GTG TGG CAA TCC AA	58–42 °C, -2 °C/3 cycles + 11 cycles @ 42 °C

#### ﻿Sequence analysis and molecular systematics

We used PhyloSuite v.1.2.3 (Zhang et al. 2020) for molecular phylogeny and tree‐based analyses. The concatenated dataset (the full dataset) contained all 56 taxa. These taxa had both the 28S and EF1α gene fragments, although some individuals were missing one gene fragment. Some genes were obtained from NCBI (Table 2), originating from Morse and Normark (2006) and Normark et al. (2019). Each gene fragment was analyzed independently in order to look for congruence between the loci, and to verify the vertically inherited nature of the endosymbiont sequences. Maximum likelihood and Bayesian analyses were run on the dataset, as well as on the individual gene fragments themselves. The concatenated datasets were partitioned as follows: 28S; EF1α 1^st^, 2^nd^, and 3^rd^ codon positions. This partition scheme was used in both the Maximum likelihood and Bayesian analyses. Each partition was examined in ModelFinder (Kalyaanamoorthy et al. 2017) to determine the most appropriate model on DNA evolution. Maximum likelihood phylogenies were inferred using IQ-TREE v.2.2.0 (Nguyen et al. 2015) under the TIMe+I+G4+F model for 5000 ultrafast (Minh et al. 2013) bootstraps. Bayesian Inference phylogenies were inferred using MrBayes v.3.2.7a (Ronquist et al. 2012) under SYM+I+G model (2 parallel runs, 1999997 generations), in which the initial 25% of sampled data were discarded as burn-in.

**Table 2.** Taxa included in the phylogenetic analysis.

Species name	Isolate	28S/GenBank accession numbers	EF-1α/GenBank accession numbers
*Aonidiellaaurantii*	D3778A	KY219911.1	KY221741.1
*Aspidiotusdestructor*	D0496A	KY219108.1	KY221360.1
*Aulacaspisalisiana*	D2435B	KY219609.1	–
	D2435A	KY219608.1	–
	D2434B	KY219606.1	–
	D2434C	KY219607.1	–
*Aulacaspiscrawii*	D3360B	KY219853.1	–
*Aulacaspisdifficilis*	D0375A	KY219047.1	KY221320.1
	D0375E	KY219048.1	KY221321.1
	D2474B	KY219626.1	KY221577.1
*Aulacaspisspinosa*	D376A	DQ145367.2	DQ145479.1
*Aulacaspisdistylii*	D0384A	KY219057.1	KY221329.1
	D0384C	KY219058.1	–
*Aulacaspisrosae*	D0395C	KY219081.1	KY221343.1
	D0395A	GQ325451.1	GQ403823.1
	D1433A	KY219387.1	KY221462.1
	D0395B	GQ325452.1	GQ403824.1
*Aulacaspisrosarum*	D1804D	KY219405.1	KY221468.1
*Aulacaspistubercularis*	D1180B	KY219359.1	KY221454.1
	D1180C	KY219360.1	–
	D0225C	KY218955.1	KY221261.1
	D0225D	KY218956.1	KY221262.1
	D2952A	KY219759.1	KY221643.1
	D1180A	KY219358.1	KY221453.1
*Aulacaspisvitis*	D4354A	KY219974.1	KY221782.1
*Aulacaspisyabunikkei*	D2472A	KY219622.1	–
*Aulacaspisyasumatsui*	D0242A	KY218963.1	KY221269.1
	D1092C	KY219318.1	–
	D5050B	KY220038.1	–
	D0992A	KY219297.1	KY221440.1
	D1093A	KY219319.1	–
	D0307A	KY219014.1	KY221302.1
	D1833A	KY219407.1	–
	D0304A	KY219011.1	KY221301.1
	D0304C	KY219013.1	–
	D0304B	KY219012.1	–
*Chionaspisamericana*	D2427A	KY219604.1	KY221572.1
	D2181A	GQ325453.1	GQ403945.1
	D0833D	KY219245.1	–
	D0833A	GQ325454.1	GQ403869.1
*Chionaspisetrusca*	D0606A	KY219146.1	KY221382.1
	D0687A	GQ325455.1	GQ403852.1
	D0687B	KY219175.1	–
	D0687C	KY219176.1	–
	D0687D	KY219177.1	KY221398.1
	D0687E	KY219178.1	KY221399.1
	D0810B	KY219234.1	KY221418.1
	D0811A	KY219235.1	KY221419.1
	D2528A	KY219649.1	–
*Chionaspisgleditsiae*	D0932A	KY219276.1	JX677928.1
*Chionaspiskosztarabi*	D1950A	KY219471.1	KY221509.1
*Chionaspisnyssae*	D1109B	KY219325.1	KY221445.1
	D0939A	KY219278.1	KY221436.1
*Chionaspisortholobis*	D2713B	KY219704.1	–
*Chionaspissalicis*	D2903A	KY219746.1	KY221633.1
	D0662A	GU349105.1	GU349853.1
	D0662B	KY219174.1	–
*Chionaspiswistariae*	D0386A	GU349092.1	GU349840.1
	D0386B	KY219061.1	–
*Duplachionaspisdisplicata*	D3506B	KY219861.1	KY221706.1
*Duplachionaspisdivergens*	D0309E	KY219016.1	–
	D0309C	GQ325478.1	GQ403821.1
	D1124B	KY219336.1	KY221446.1
	D2863A	KY219741.1	–
	D1125B	KY219337.1	–
	D0309B	KY219015.1	KY221303.1
*Duplachionaspissicula*	D0623C	KY219156.1	KY221389.1
	D0623B	KY219155.1	KY221388.1
	D0623A	GU349091.1	GU349839.1
*Duplachionaspisspartinae*	D2909A	KY219747.1	KY221634.1
	D2442A	KY219612.1	–
*Megacanthaspisleucaspis*	D0401A	KY219088.1	KY221347.1
	D0401E	KY219089.1	–
*Pinnaspisaspidistrae*	D2535A	KY219653.1	–
	D2537A	KY219654.1	–
	D2939A	KY219753.1	KY221638.1
*Pinnaspishikosana*	D0388D	KY219065.1	–
*Pinnaspisstrachani*	D0248A	KY218966.1	KY221272.1
	D0248C	KY218967.1	KY221273.1
*Pseudaulacaspispentagona*	D0372A	KY219042.1	KY221316.1
*Superturmaspisschizosoma*	–	OQ917119	OQ869250
	–	OQ917120	OQ869251

## ﻿Taxonomy

### ﻿ Aulacaspis

Taxon classificationAnimaliaHemipteraDiaspididae

Cockerell

8C21FA98-2D19-5036-83EC-414BC81C3856

Aulacaspis Cockerell, 1893: 180.

#### Type species.

*Aspidiotusrosae* Bouché: by subsequent designation by Newstead 1901: 168.

#### Generic diagnosis.

The following diagnosis is taken from Miller and Davidson (2005) and Takagi (2012). ***Female scale.*** White, circular, exuviae located on anterior end. ***Male scale.*** White, long, and narrow, exuviae located on anterior end. ***Adult female.*** Body elongate; derm membranous except for the margin of pygidium; prosoma generally swollen or wider than metathorax and abdomen, slightly squared in most species. ***Cephalothorax.*** Antennae each with a seta. Anterior spiracles usually with a cluster of trilocular pores, posterior spiracles with or without associated trilocular pores. Dorsal ducts present or absent on prosoma, scattered. ***Pygidium.*** Usually with three pairs of lobes (rarely with two or four pairs). Median lobes (L1) well developed, sunken into or protruding apex of pygidium, much larger than lobules of lateral lobes, zygotic basally or not, without marginal setae between lobes. In general, L1 are divided into two types depending on feeding site: bark-type, where individuals occur on bark and L1 protrudes at the end of the pygidium; and leaf-type, on leaves and L1 is sunken into the end of pygidium. Second lobes (L2) much smaller than L1, bilobed, divided into inner lobule and outer lobule, outer lobule usually smaller than inner lobule. Third lobes (L3) smaller than L2, bilobed, outer lobule smaller than inner lobule. Fourth lobes (L4) present in some species and usually represented by serrations along the body margin. ***Gland spines.*** Marginal gland spines developed, present on lateral of abdominal segment II and III; usually single on abdominal segments V–VIII, but in some species two or more. Marginal gland spines becoming shorter to conical on anterior segments; in some species they are called gland tubercles. ***Ducts.*** Dorsum with double-barred ducts. Marginal macroducts of pygidium usually larger than dorsal macroducts. Dorsal macroducts forming submedial and submarginal rows on abdominal segment and pygidium, sometimes occurring in two sizes. Dorsal ducts on the lateral margins of the second and the third abdominal segments. Ventral microducts scattered. Anal opening situated at the center of the pygidium, small. Perivulvar disc pores in five groups.

#### Remarks.

As in other members of the subtribe Chionaspidina, *Aulacaspis* species have median lobes often joined by a zygosis; without setae or gland spines or marginal macroducts between median lobes (Miller and Davidson 2005; Takagi 2012). *Aulacaspis* is distinguished from other genera, especially from *Chionaspis* and *Myrtaspis* Takagi, 1999 by lacking lateral macroducts and gland spines on abdominal segment I and on the thorax, present in these locations on *Chionaspis* and *Myrtaspis* (Takagi 1999).

### ﻿ Aulacaspis fanjingshanensis sp. nov.

Taxon classificationAnimaliaHemipteraDiaspididae

F5661EDB-A7EC-5C82-80D1-85561BCC7DD7

https://zoobank.org/9A000A90-B394-4409-A75D-F846C571BDAC

Figs 1–4

#### Material examined.

**Holotype female**: China, Guizhou Province, Tongren city, Fanjing Mountain, on leaves of an undetermined plant of the family Rosaceae, coll. Wei Jiufeng and Niu Minmin, 8.viii.2015 (at SXAU). **Paratypes female**: CHINA, same data as holotype (at SXAU; a total of 2 adult females, 2 slides each containing 1 adult females).

**Figures 1–4.**
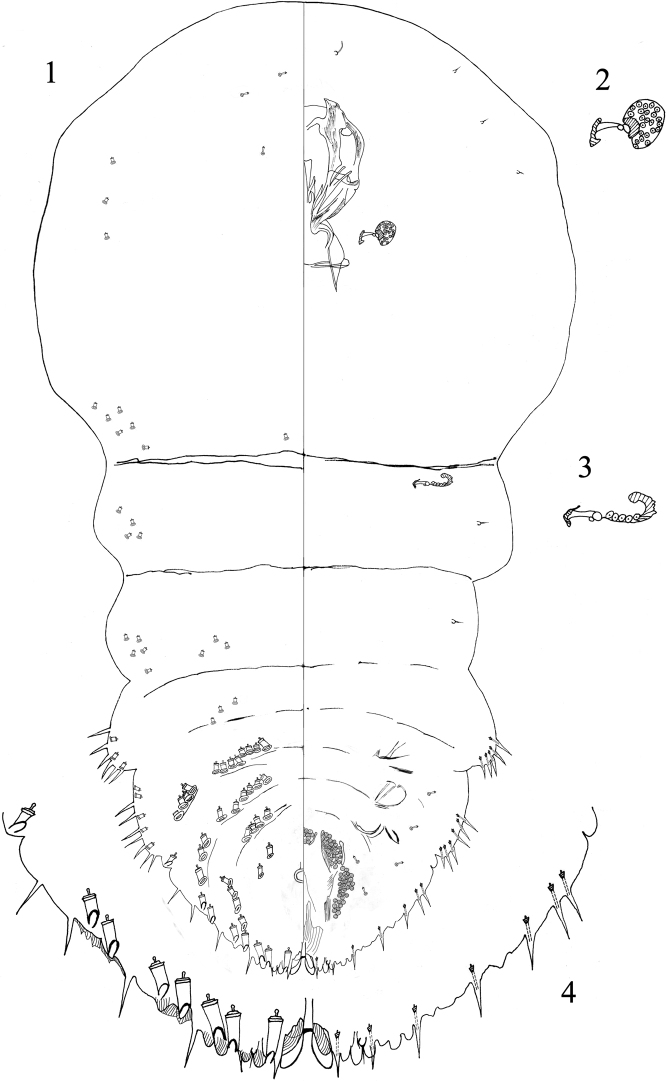
*Aulacaspisfanjingshanensis* Niu & Wei, sp. nov., adult female **1** body **2** anterior spiracle **3** posterior spiracle **4** margin of pygidium.

#### Description of slide-mounted adult females.

***Prosoma.*** Prosoma with anterior margin rounded and with sides nearly parallel; peribuccal scleroses well formed. Antennae separated by 60–67 µm; each antenna with 1 seta. Anterior spiracles each associated with 24–27 trilocular disc pores; posterior spiracles each with 3–7 disc pores. ***Pygidial lobes.*** With 3 pairs of lobes; L1 well developed, largely sunken into apex of pygidium, base of each L1 with a mesad linear extension onto ventral derm, these extensions separated from each other by a narrow space; L1 elongate and divergent, widest separation distance 19–22 µm, divergent mesal margins minutely serrate, lobe apices blunt or rounded. Setae absent from between median lobes. L2 and L3 well developed, bilobulate. Marginal macroducts, of two-barred type, nearly as long as L1, absent between L1, one present between L1 and L2, two present between L2 and L3, two present on the abdominal segment V. Dorsal macroducts on pygidium and abdominal segments shorter than marginal macroducts; of two-barred type, arranged segmentally in submedian and submargin rows; submarginal dorsal macroducts present on abdominal segment III to V: 4–9 on segment III, 1–6 on segment IV, 2–5 on segment V; submedian dorsal macroducts present on segment III to VI, on III and IV being divided into segmental and infrasegmental series: front row 4, rear row 3–4 on segment III, front row 4–5, rear row 1–3 on segment IV, 2–5 on segment V, 2–3 on segment VI. Dorsal microducts present on abdominal segment II to head: 5 on forehead, 2–4 on the position of mouthparts, 3–4 on the submargin of prothorax, 5–6 on the submargin of mesothorax, 1 on the middle area of mesothorax, 4 on the submargin area of metathorax, 3 on the submedian area of segment I, 5 on the submargin area of segment I, 2–3 on the submedian area of segment II, 0–2 on the submargin area of segment II. Lateral ducts few, 5–7 in total, present between abdominal area of segments II and III, 2–4 on segment II, 2–5 on segment III, smaller than dorsal ducts present on abdominal segments and pygidium. Ventral microducts scattered on pygidium, few. Marginal gland spines on each side numbering 2‒4 on segment IV, and 1 on V; also, with 1 lateral to each pygidial lobe. Each side with lateral gland spines numbering 5‒7 on segment II, and 6–13 on segment III. Perivulvar pores in five groups, with 10–17 in the median group, 20–26 in each anterolateral group and 27–35 in each posterolateral group.

#### Host.

Rosaceae sp.

#### Etymology.

The specific epithet is formed by a combination of Fanjing Mountain, the type locality, and the Latin “-ensis”, meaning “from”.

#### Distribution.

China (Guizhou Province).

#### Remarks.

*Aulacaspisfanjingshanensis* sp. nov. is similar to other species in the *Yabunikkei* complex (*A.yabunikkei* Kuwana, 1926, *A.shirodamo* Takagi, 2014 and *A.neolitseae* Takagi, 2014) by the form of its body shape, but can be distinguished from *A.yabunikkei* by the following characteristics of the adult female (character-states of *A.yabunikkei* in brackets): (1) dorsal microducts present on abdominal segment II to head (absent); (2) marginal macroducts of abdominal segment VII scarcely or only a little extending anteriorly beyond bases of these extensions (extending); and (3) median lobes separated from each other by a narrow space (united basally).

The stable diagnostic characters that distinguish *A.fanjingshanensis* sp. nov. from *A.shirodamo* and *A.neolitseae* are very few. The differences are the presence of dorsal microducts that are found from the head region down to abdominal segment II and median lobes separated from each other by a narrow space.

### ﻿Key to adult female *Aulacaspis* Cockerell from Guizhou Province of China

**Table:** 

1	Abdominal segment I with dorsal microducts or macroducts	**2**
–	Abdominal segment I without dorsal microducts or macroducts	**3**
2	Abdominal segment I with dorsal microducts	***Aulacaspisfanjingshanensis* sp. nov.**
–	Abdominal segment I with dorsal macroducts	***A.crawii* (Cockerell)**
3	Abdominal segment VI without dorsal macroducts	***A.zunyiensis* Wei**
–	Abdominal segment VI with dorsal macroducts	**4**
4	Abdominal segment II with submedial dorsal macroducts	**5**
–	Abdominal segment II without submedial dorsal macroducts	**7**
5	Prosomatic tubercles prominent, prosoma angular	***A.guiyangensis* Tian & Xing**
–	Prosomatic tubercles not developed, prosoma suborbicular	**6**
6	Lateral gland spines many, numbering about 30–45 on each side	***A.paralonganae* Tian & Xing**
–	Lateral gland spines few, numbering about 10–25 on each side	***A.longanae* Chen, Wu & Su**
7	Submarginal and submedial dorsal macroducts all arranged in irregularly double or triple rows	***A.saigusai* Takagi**
–	Submedial dorsal macroducts arranged in double rows	***A.yabunikkei* Kuwana**

### ﻿ Superturmaspis schizosoma

Taxon classificationAnimaliaHemipteraDiaspididae

(Takagi, 1970), revived combination

926CE5B3-6F24-515B-9D2E-B043F4B09A72

Chionaspis schizosoma Takagi, 1970: 77.
Superturmaspis schizosoma (Takagi, 1970); Chen 1983: 86. Change of combination.
Aulacaspis schizosoma (Takagi, 1970); Takagi 1985: 49. Change of combination.
Semichionaspis schizosoma (Takagi, 1970); Tang 1986: 170. Change of combination.
Aulacaspis schizosoma (Takagi, 1970); Takagi 1999: 149. Revived combination.

#### Material examined.

China, Zhejiang Province, Taohua Island in Zhoushan Islands, on Lauraceae leaves, coll. Niu Minmin, 31.v.2017 (at NWAFU).

#### Remarks.

We confirmed the non-monophyly of the genus-level relationships and taxonomy of Chionaspidina, including the genera *Aulacaspis* and *Chionaspis* (Normark et al. 2019). The same level of strong statistical support was observed for the species in our two molecular phylogenetic trees (ML and BI trees, Figs 6, 7). On those phylogenetic trees, *Superturmaspisschizosoma* comprises a clade separate from the one that includes the genera *Aulacaspis*, *Chionaspis*, *Duplachionaspis*, *Pinnaspis* and *Megacanthaspis* (In ML tree, the bootstrap value is 52, and in BI tree, the posterior probability is 1).

Our phylogenetic study has demonstrated that the type species of *Aulacaspis* and *Chionaspis* (*A.rosae* and *C.salicis*) are more closely related to each other than either is to *Superturmaspisschizosoma*. Hence, *A.schizosoma* was wrongly assigned to *Aulacaspis* (Figs 6, 7), although the species *Superturmaspisschizosoma* (Takagi, 1970) is morphologically very close to *Aulacaspis*, sharing very similar characters of the pygidial margin and a similar distribution of dorsal ducts, but differing by being widest across the mesothorax, body of adult female is top form in outline, pronounced contracted segmentation on lateral margins which contains the cephalothorax and abdominal segments preceding the pygidium.

**Figure 5.**
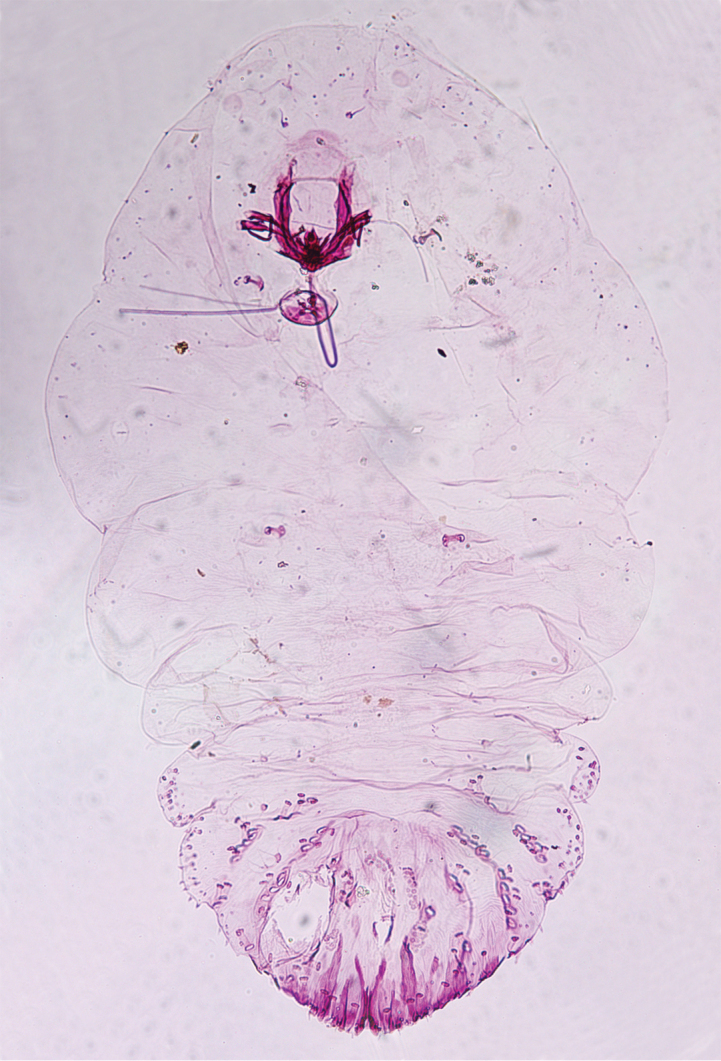
*Superturmaspisschizosoma* (Takagi, 1970), revived combination, adult female.

We transferred the species back to *Superturmaspis* based on our molecular analysis. *Superturmaspis* was previously established by Chen, 1983, and included only one species, *Superturmaspisschizosoma*. The adult female specimens (Fig. 5) substantially agree with the type-series from Taiwan (Takagi 1970). The body of the adult female is robust at maturity, spinning top form in outline, widest across the mesothorax, pronouncedly contracted segmentation on lateral margins which contains the cephalothorax and abdominal segments preceding the pygidium, and marginal macroducts rather stout and long.

**Figure 6.**
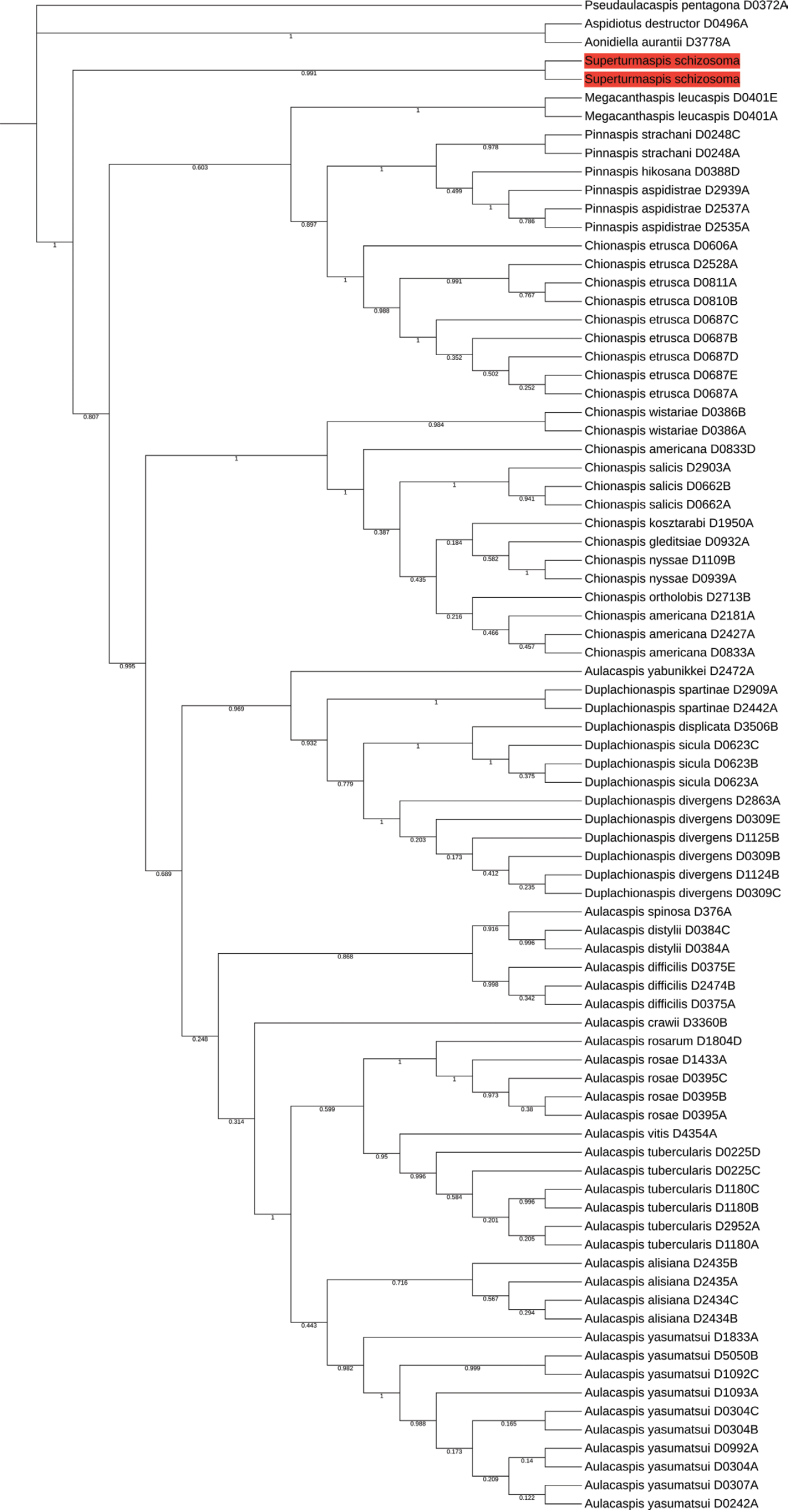
The ML tree based on the concatenated data of 28S and EF-1α. Bootstrap values (BV) are shown on each node. Orange shading represents the position of *Superturmaspisschizosoma* (Takagi, 1970) on the phylogenetic tree.

**Figure 7.**
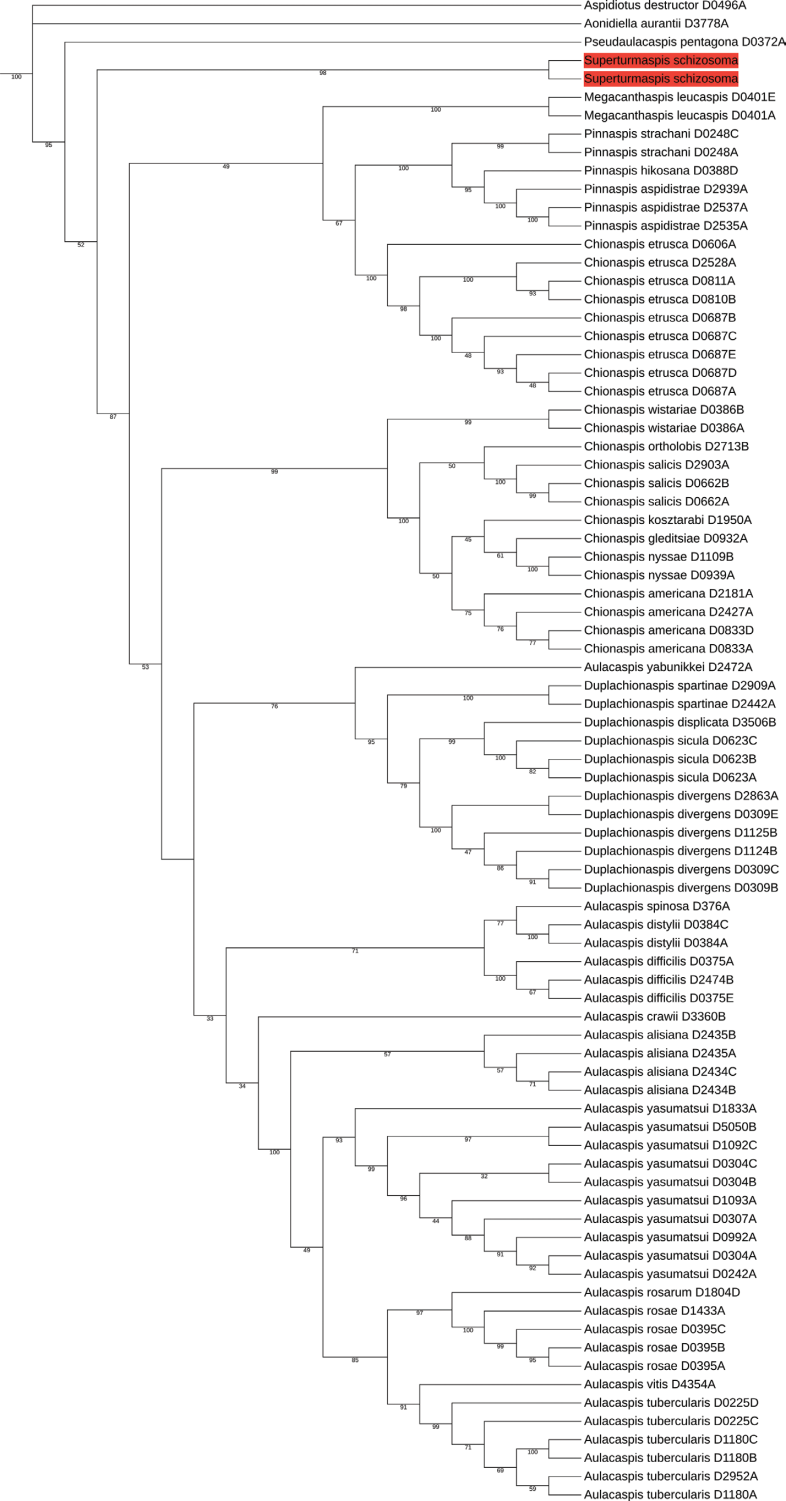
The BI tree based on the concatenated data of 28S and EF-1α. Posterior probabilities (pp) are shown on each node. Orange shading represents the position of *Superturmaspisschizosoma* (Takagi, 1970) on the phylogenetic tree.

## Supplementary Material

XML Treatment for Aulacaspis

XML Treatment for Aulacaspis fanjingshanensis

XML Treatment for Superturmaspis schizosoma

## ﻿Additional information

### Author ORCIDs

Minmin Niu https://orcid.org/0009-0004-6506-4950

### Data availability

All of the data that support the findings of this study are available in the main text.

## References

[B1] Andersen JC Wu J Gruwell ME Gwiazdowski R Santana SE Feliciano NM Morse GE Normark BB (2010) A phylogenetic analysis of armored scale insects, based upon nuclear, mitochondrial, and endosymbiont gene sequences. Molecular Phylogenetics and Evolution 57: 992‒1003. 10.1016/j.ympev.2010.05.002 20460159

[B2] Bouché PF (1833) Naturgeschichte der Schädlichen und Nützlichen Garteninsekten und die bewährtesten Mittel zur Vertilgung der ersteren. Berlin In der Nicolaischen Buchhandlung, 176 pp. 10.5962/bhl.title.9692

[B3] Chen FG (1983) The Chionaspidini (Diaspididae, Coccoidea, Homoptera) from China. Science & Technology Publishing House, Sichuan Province, China, 175 pp.

[B4] Cockerell TDA (1893) Museum notes, Coccidae. Journal of the Institute of Jamaica 1: 180.

[B5] Easton ER Pun WW (1999) Observations on twelve families of Homoptera in Macau, Southeastern China, from 1989 to the present. Proceedings of the Entomological Society of Washington 101(1): 99–105.

[B6] Ferris GF (1936) Contributions to the knowledge of the Coccoidea (Homoptera). II. (Contribution no. 2). Microentomology 1: 17–92.

[B7] García Morales M Denno B Mille DR Miller GL Ben-Dov Y Hardy NB (2016) ScaleNet: a literature-based model of scale insect biology and systematics. http://scalenet.info [Accessed 22 November 2022] 10.1093/database/bav118 PMC4747323 26861659

[B8] Gruwell ME Morse GE Normark BB (2007) Phylogenetic congruence of armored scale insects (Hemiptera: Diaspididae) and their primary endosymbionts from the phylum Bacteroidetes. Molecular Phylogenetics and Evolution 44: 267‒280. 10.1016/j.ympev.2007.01.014 17400002

[B9] Henderson RC (2011) Diaspididae (Insecta: Hemiptera: Coccoidea). Fauna of New Zealand 66. Manaaki Whenua Press, Lincoln, Canterbury, 275 pp.

[B10] Hua LZ (2000) List of Chinese Insects (Vol. 1). Zhongshan University Press Guangzhou, China, 448 pp.

[B11] Kalyaanamoorthy S Minh BQ Wong TKF von Haeseler A Jermiin LS (2017) ModelFinder: Fast model selection for accurate phylogenetic estimates. Nature Methods 14(6): 587–589. 10.1038/nmeth.4285 28481363 PMC5453245

[B12] Kuwana SI (1926) The diaspine Coccidae of Japan. IV. Genera *Cryptoparlatoria*, *Howardia*, *Sasakiaspis* [n. gen.] *Diaspis*, *Aulacaspis*, *Pinnaspis* and *Prontaspis*. Department of Agriculture and Commerce, Bureau of Agriculture. Injurious Insects and Pests, Japan 4: 1–44.

[B13] Miller DR Davidson JA (2005) Armored Scale Insect Pests of Trees and Shrubs (Hemiptera: Diaspididae). Cornell University Press, Ithaca, 456 pp.

[B14] Minh BQ Nguyen MA von Haeseler A (2013) Ultrafast approximation for phylogenetic bootstrap. Molecular Biology and Evolution 30(5): 1188–1195. 10.1093/molbev/mst024 23418397 PMC3670741

[B15] Morse GE Normark BB (2006) A molecular phylogenetic study of armoured scale insects (Hemiptera: Diaspididae). Systematic Entomology 31(2): 338–349. 10.1111/j.1365-3113.2005.00316.x

[B16] Newstead R (1901) Monograph of the Coccidae of the British Isles. Ray Society London, 220 pp. 10.5962/bhl.title.21716

[B17] Nguyen LT Schmidt HA von Haeseler A Minh BQ (2015) IQ-TREE: A fast and effective stochastic algorithm for estimating maximum-likelihood phylogenies. Molecular Biology and Evolution 32(1): 268–274. 10.1093/molbev/msu300 25371430 PMC4271533

[B18] Normark BB Okusu A Morse GE Peterson DA Itioka T Schneider SA (2019) Phylogeny and classification of armored scale insects (Hemiptera: Coccomorpha: Diaspididae). Zootaxa 4616(1): 1–98. 10.11646/zootaxa.4616.1.1 31716328

[B19] Ronquist F Teslenko M van der Mark P Ayres DL Darling A Höhna S Larget B Liu L Suchard MA Huelsenbeck JP (2012) MrBayes 3.2: Efficient Bayesian phylogenetic inference and model choice across a large model space. Systematic Biology 61(3): 539–542. 10.1093/sysbio/sys029 22357727 PMC3329765

[B20] Takagi S (1970) Diaspididae of Taiwan based on material collected in connection with the Japan-U.S. Cooperative Science Programme, 1965 (Homoptera: Coccoidea). Pt. II. Insecta Matsumurana 33: 1–146.

[B21] Takagi S (1985) The scale insect genus *Chionaspis*: A revised concept (Homoptera: Coccoidea: Diaspididae). Insecta Matsumurana New Series 33: 1–77.

[B22] Takagi S (1999) For a better understanding of *Aulacaspis*: The *calcarata* species group (Homoptera: Coccoidea: Diaspididae). Insecta Matsumurana New Series 55: 133–180.

[B23] Takagi S (2012) Atypical species of *Aulacaspis* (Sternorrhyncha: Coccoidea: Diaspididae). Insecta Matsumurana New Series 68: 17–115.

[B24] Tang FT (1986) The scale insects of horticulture and forest of China, Volume III. Shanxi Agricultural University Press Taigu, Shanxi, 305 pp.

[B25] Tian F Xing JC (2022) Two new species of *Aulacaspis* Cockerell, 1893 (Hemiptera: Coccomorpha: Diaspididae) from China. Zootaxa 5087(1): 154–178. 10.11646/zootaxa.5087.1.7 35390921

[B26] Wei JF Jing XP Zhang HF (2016) A new species of *Aulacaspis* Cockerell, 1893 from China with a key to Chinese species (Hemiptera, Coccoidea, Diaspididae). ZooKeys 619: 13–24. 10.3897/zookeys.619.9399 PMC5090160 27829787

[B27] Zhang D Gao F Jakovlić I Zou H Zhang J Li WX Wang GT (2020) PhyloSuite: An integrated and scalable desktop platform for streamlined molecular sequence data management and evolutionary phylogenetics studies. Molecular Ecology Resources 20(1): 348–355. 10.1111/1755-0998.13096 31599058

